# Supplementary material for: Reimagining Microbially Induced Concrete Deterioration: A Novel Approach Through Coupled Confocal Laser Scanning Microscope–Avizo Three-Dimensional Modeling of Biofilms
Source: Microorganisms. 2025 Jun 23;13(7):1452. doi: 10.3390/microorganisms13071452 (PMC12301018; doi:10.3390/microorganisms13071452)
Supplement: Supplementary file 1 [file microorganisms-13-01452-s001.zip › Table S1.pdf]

Chemical composition of Portland cement (PC), ground granulated blast furnace slag (GBFS), basalt powder (BP), and desulfurized gypsum (DG).

No.	Chemical composition (%)								Physical properties	
	SiO_2	Al_2O_3	Fe_2O_3	CaO	MgO	SO_3	Na_2O	K_2O	SG[a]	SSA[a]
PC	19.4	4.9	4.01	61.9	1.93	3.09	0.63	0.67	3.04	357.4
GBFS	34.2	11.6	0.65	39.9	6.94	2.34	0.67	0.96	2.86	479.7
BP	49.8	15.4	11.3	9.16	4.40	0.21	3.86	1.51	2.82	242.8
DG	2.22	0.56	0.19	33.8	1.14	48.3	0.13	0.10	2.33	141.2

[a] Note: SG, specific gravity; SSA, specific surface area (m^2/kg).
