# Supplementary figures and images for: Reimagining Microbially Induced Concrete Deterioration: A Novel Approach Through Coupled Confocal Laser Scanning Microscope–Avizo Three-Dimensional Modeling of Biofilms
Source: Microorganisms. 2025 Jun 23;13(7):1452. doi: 10.3390/microorganisms13071452 (PMC12301018; doi:10.3390/microorganisms13071452)

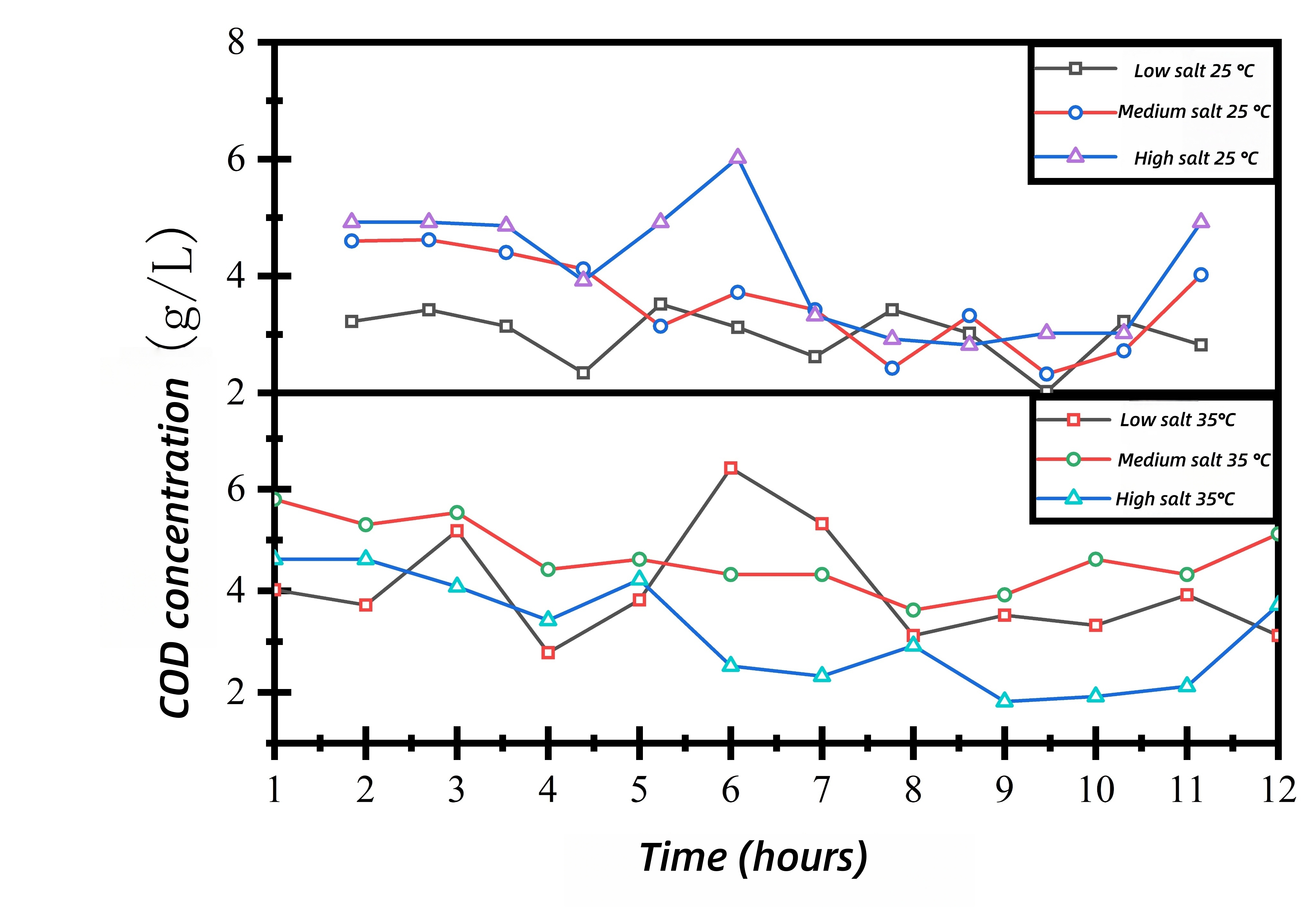

Supplement: Supplementary file 1 [file microorganisms-13-01452-s001.zip › Figure S1.jpg]

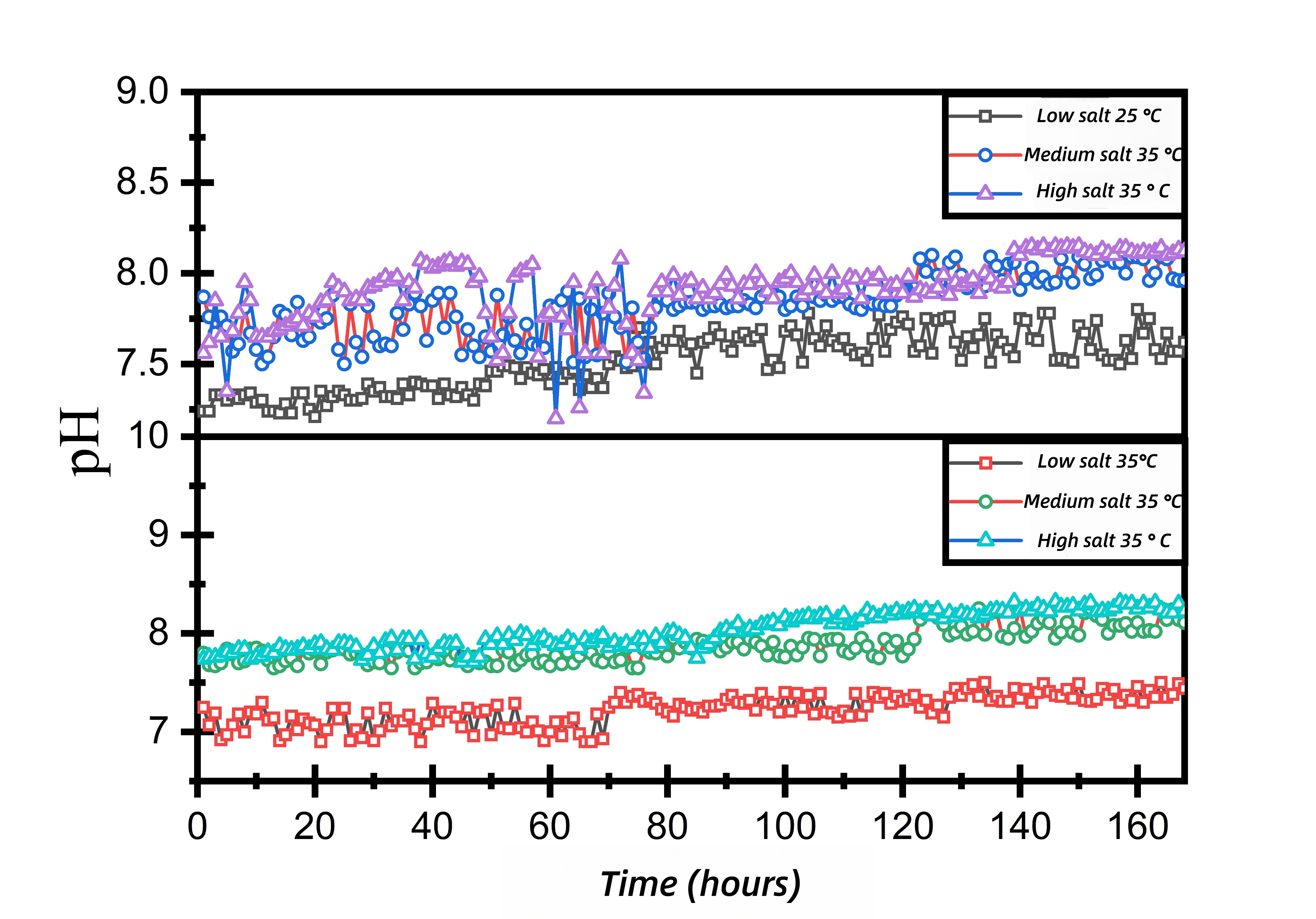

Supplement: Supplementary file 1 [file microorganisms-13-01452-s001.zip › Figure S2.jpg]

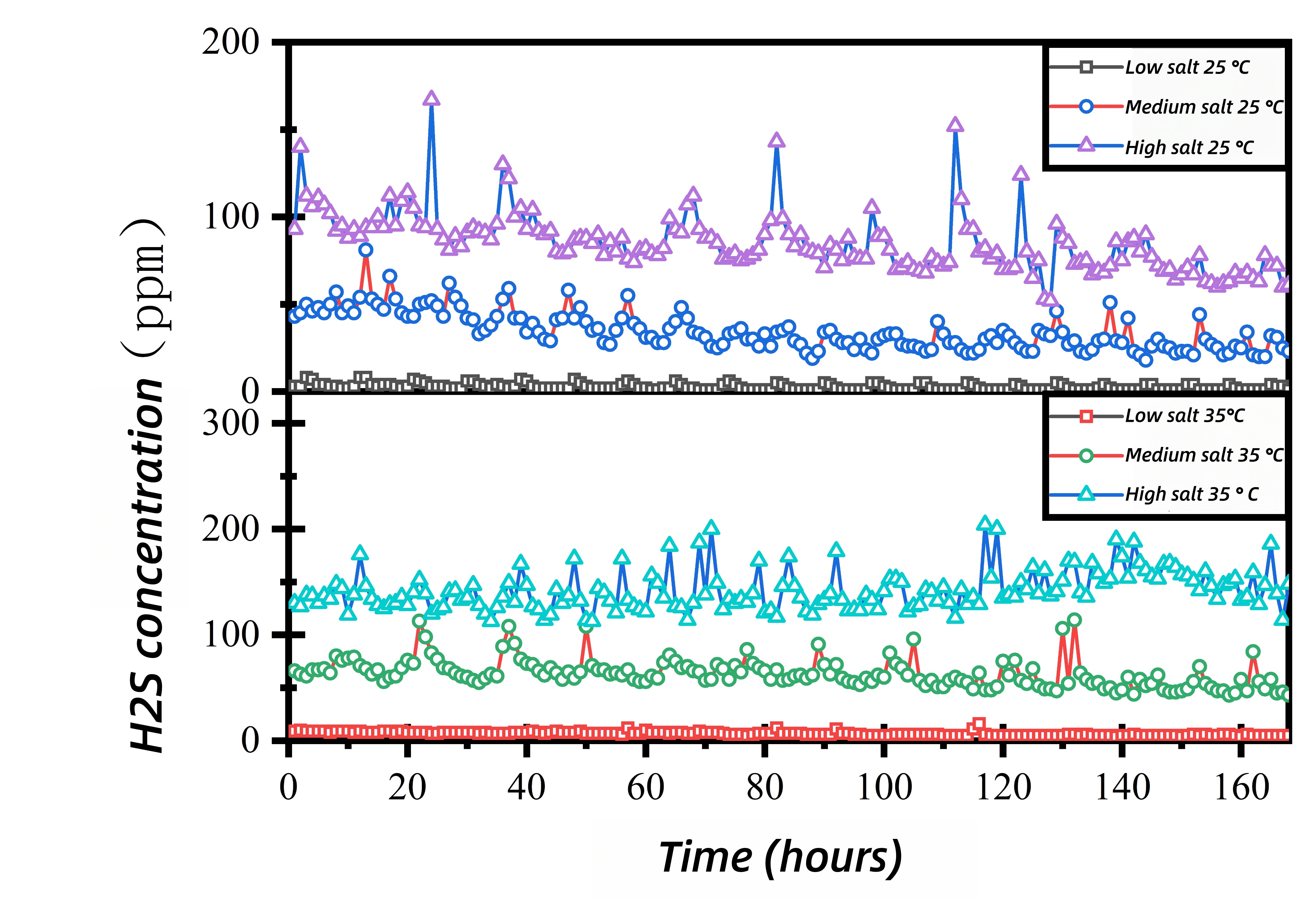

Supplement: Supplementary file 1 [file microorganisms-13-01452-s001.zip › Figure S3.jpg]

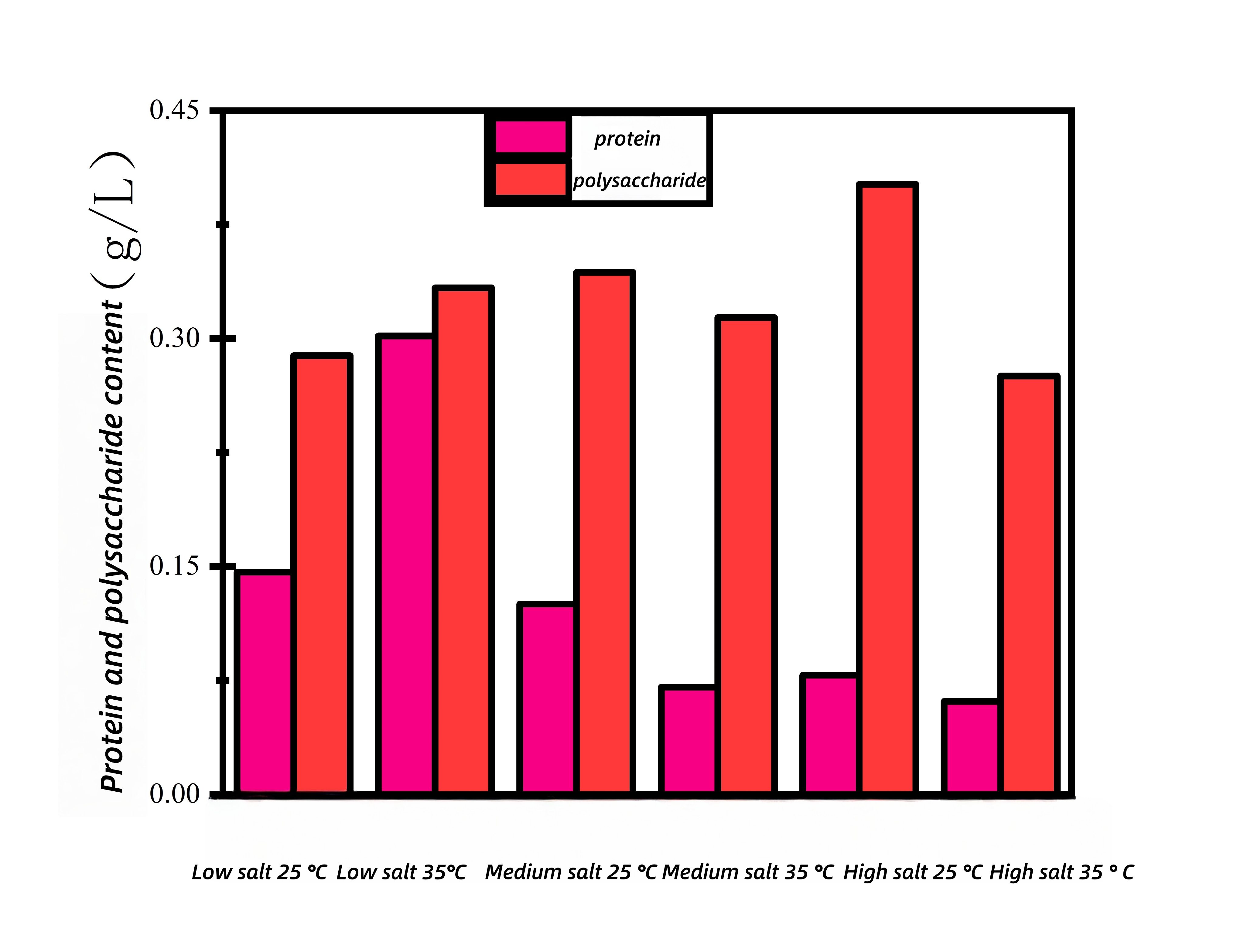

Supplement: Supplementary file 1 [file microorganisms-13-01452-s001.zip › Figure S4.jpg]
